# Intervention using a novel biodegradable hollow stent containing polylactic acid-polyprolactone-polyethylene glycol complexes against lacrimal duct obstruction disease

**DOI:** 10.1371/journal.pone.0178679

**Published:** 2017-06-01

**Authors:** Xinyuan Zhan, Xin Guo, Rong Liu, Weikun Hu, Lei Zhang, Nan Xiang

**Affiliations:** 1 Department of Anesthesiology, Tongji Hospital, Tongji Medical College, Huazhong University of Science and Technology, Wuhan, China; 2 Department of Orthopedics, Puai Hospital, Tongji Medical College, Huazhong University of Science and Technology, Wuhan, China; 3 Department of Ophthalmology, Tongji Hospital, Tongji Medical College, Huazhong University of Science and Technology, Wuhan, China; 4 College of Chemistry and Chemical Engineering, Huazhong University of Science and Technology, Wuhan, China; Universita degli Studi di Palermo, ITALY

## Abstract

Lacrimal duct obstruction disease (LDOD) is a common ophthalmologic disease. Stent implantation surgery is one of the most effective therapies. In this study, we intended to find out the satisfactory biodegradable stents containing poly-L-lactic acid-polycaprolactone-polyethylene glycol (PLLA- PCL- PEG) complexes for therapeutic application in LDOD. Stents made of PLLA- PCL- PEG complexes in various ratios, were prepared and used *in vitro* to determine stents with appropriate mechanical properties and shorter range of bio-degradation for study *in vivo*. Thirty-two rabbits were randomized into eight groups of four eyes each in advance for test *in vivo*. The selected stents were implanted into the left lacrimal ducts of 16 rabbits and silica gel stents as the control for the other 16 rabbits. At four points in time (1, 4, 10 and 16 weeks after the implantation), weight loss rate (WLR) of the stents was measured and analysed. To access the change of lacrimal duct, fluorescein excretion test, lacrimal duct endoscopy and histopathological testing were conducted. The stent containing PLLA: PCL6: 4+ 15%PEG was selected for study *in vivo*. Analysis of weight loss rate (WLR), fluorescein excretion test, lacrimal duct endoscopy and histopathological testing indicated that the selected stent was biodegradable and caused minimal stimulation and earlier tissue restoration in the lacrimal epithelium compared with the silica gel stent used as the control. The study results suggest that the PLLA: PCL6: 4+ 15% PEG stent is a satisfactory biodegradable stent as a promising alternative for therapeutic application in LDOD, which showed tissue compatibility, biodegradation and adequate mechanical intensity.

## Introduction

Lacrimal duct obstruction disease (LDOD), an illness characterized by epiphora and suppuration is a common ophthalmologic disease leading to chronic dacryocystitis unless controlled promptly. Surgery for LDOD is effective for the treatment of lacrimal ducts, and involves lacrimal duct intubation, dacryocystorhinostomy (DCR) and lacrimal bypass surgery. It is generally believed that stenting of lacrimal ducts improves curative effects and increases the success rate of surgery irrespective of the type of surgery [1, 2].

The materials constituting the stent ensure open ducts and decrease the complication rate [2, 3]. However, non-biodegradable stents lead to lacrimal duct reobstruction and epithelial fibrosis following retention in the duct and adhesion to secretions[4]. Therefore, the stent is removed surgically 1 to 3 months after the implantation. The second surgery not only increases patient’s suffering but also damages the epithelium further. As a result, removal of the classic lacrimal duct stents is a controversial intervention [5]. Even though the hollow silicone stent is regarded as one of the most effective devices used clinically, reobstruction and epithelial fibrosis post-insertion are not rare [5]. Biodegradable lacrimal duct stents represent an effective and safe alternative. The stents degrade after the operation for LDOD.

**Fig 1:**
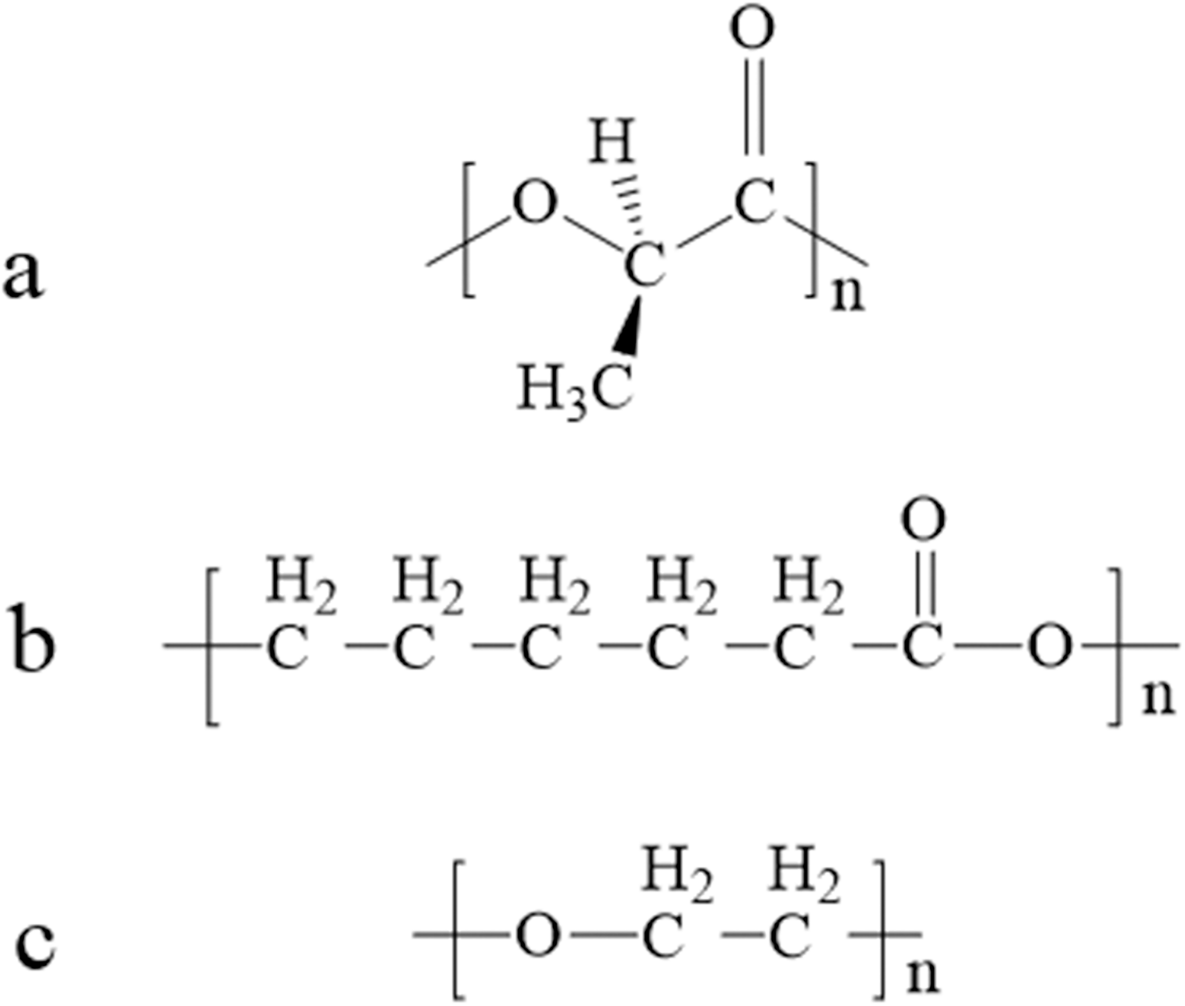
Chemical structure of PLLA (a); PCL (b); PEG (c).

Poly-L-lactic Acid (PLLA), polycaprolactone (PCL) and polyethylene glycol (PEG) (Fig 1) are three generally used biodegradable materials [6, 7] approved for clinical application by the United States Food and Drug Administration (FDA). None of the stents containing the three materials, respectively, is fit for lacrimal duct support not only because of the remodeling challenges but also due to unsatisfactory degradation. Pure PLLA stents degrade after more than one year and pure PCL and PEG stents are too weak to support the lacrimal duct. However, Korpel et al demonstrated that PLLA stents were biocompatible, based on studies involving the bronchus of rabbits [8, 9]. Further study by Ng AH [10] suggested that the PLLA bronchus stents could be remodeled easily and biodegraded rapidly when PEG was combined with PLLA. We hypothesized that PLLA would show similar effect in the lacrimal duct, which was histologically similar to bronchial mucosa. Consequently, we developed PLLA- PCL- PEG complexes in different proportions and tested their bio-degradability as well as mechanical properties *in vitro*. The stents containing complexes of biodegradable components in appropriate proportion were implanted into the lacrimal ducts of rabbits to investigate biocompatibility, biodegradation and mechanical properties for therapeutic application against LDOD.

## Materials and methods

### Preparation of biodegradable blends and stents

To determine the most appropriate PLLA- PCL- PEG complex, we tested six different PLLA- PCL ratios (100:0, 80:20, 70:30, 60:40, 50:50, 40:60) and impregnated them with 5% and 15% PEG, respectively. Toward this end, the PLLA was dried in vacuum at 70°C for 48 h and PCL and PEG were dried at 35°C for 48 h. A high-speed mixer was used to combine the complexes at 100 rpm for 6 min. The complexes were pelleted using a twin-screw melt extruder (SHJ-36 Nanjing GIANT Machinery co., LTD). The pellets were squeezed into 70×50×1 mm chips for examination.

**Fig 2:**
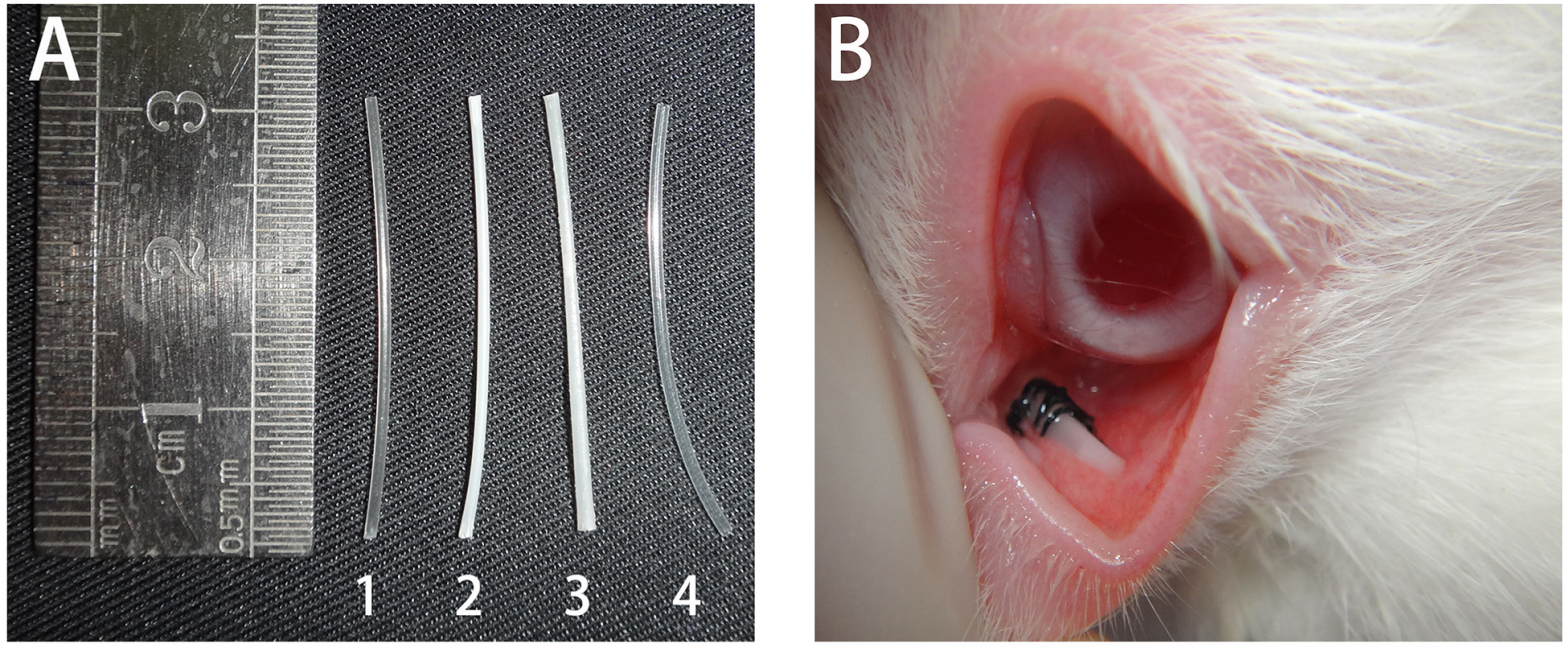
**Four representative stents designed for lacrimal duct implantation are shown in Image A.** The pure PLLA stent, PLLA; PCL6:4 stent; PLLA: PCL6:4 + 15% PEG; and silica gel stent correspond to the numbers below: 1, 2, 3 and 4. All of them showed smooth surface and hollow configuration. Image B shows the location and mechanism of lacrimal stent fixation at the lacrimal duct after implantation.

To prepare hollow stents for implantation, pellets were dried in vacuum and melted at 130–160°C. They were squeezed out of the single screw extrusion machine (SH-30 Nanjing GIANT Machinery co., LTD) and remodeled into hollow cylinders. All of the pipes were 30 mm in length, 0.8 to 1.1 mm in diameter and 0.14–0.38 mm in thickness, with slightly curved shape and a smooth end (Fig 2A).

### Mechanical properties

Impact strength indicates toughness of materials. The index of the PLLA- PCL- PEG blends at different ratios was evaluated in a Charpy impact strength test for rigidity using a GB/T 1043–93 standard impact elasticity tester (ZBC-1400-1, Xinsansi material co.). The glass transition temperature (T_g_), an important index indicating plasticity of polymers, was evaluated. A Dynamic Mechanical Analyzer (Q800, TA Technology co.) was used to analyze PLLA- PCL- PEG complexes measuring 35×10×1mm under the following conditions: single cantilever model, frequency 1 Hz, amplitude of vibration 15 μm, and 5°C/min from 25°C to 100°C. Testing of randomly selected samples at each ratio decreased the possible error.

### Biodegradability of stents in vitro

Twenty stents at different PLLA- to- PCL ratios (100: 0, 80:20, 70:30, 60:40 with PEG 5% and 15%), respectively, were selected randomly and weighed as (W1). Each of them was transferred to an Eppendorf tube with 0.1 M Phosphate Buffer Saline (PBS) at 37°C for three months to simulate the degradation process in vivo. They were removed at different intervals of time: 4 weeks, 10 weeks and 16 weeks after soaking, and rinsed three times with double-distilled water and dried in a vacuum drying box under 35°C for 24 h. The weight loss rate (WLR) was calculated by comparing the W2 with W1 as follows: WLR = (W1-W2)/W1.

Complexes of PLLA- PCL 50:50 and 40:60 with PEG 5% and 15% respectively were not included due to technical reasons as well as PLLA- PCL- PEG with higher PEG proportion than 15%. These complexes became too flexible to shaping into hollow stents in the melt extruder when the proportion of PCL and PEG rising. The stent wall deformed and the lumen collapsed.

### Animals and ethical statements

The study included 32 unaffected Japanese White Rabbits, each aged 3 to 4 months and weighting approximately 2.5 ± 0.5 kg. The Specific Pathogen-Free Animal Laboratory of Tongji hospital, Wuhan supplied all the rabbits. The animals were housed individually. They were fed similar amounts of standard laboratory rabbit chow daily with water *ad libitum*. The rabbits were divided into 8 groups (A_1_, A_2_, A_3_ A_4_, B_1_, B_2_, B_3_ B_4_) randomly with 4 rabbits each. PLLA- PCL- PEG stents with the most satisfactory ratio (PLLA: PCL 6: 4+ 15% PEG) were implanted into the left lacrimal ducts (the corresponding right ducts served as blank controls) of rabbits in Groups A (A_1_, A_2_, A_3_ A_4_) and B (B_1_, B_2_, B_3_, B_4_), which served as the control groups implanted with silica gel hollow stents of the same size. NIH guidelines for the care and use of laboratory animals (NIH Publication #85–23 Rev. 1996) have been observed. The protocol was approved by the Committee on the Ethics of Animal Experiments of Tongji hospital, Wuhan. All surgery was performed under sodium pentobarbital anesthesia, and all efforts were made to minimize suffering. During the study, no rabbits were dead or severely injured prior to the experimental endpoint.

### Stent implantation

Before implantation, an intramuscular gluteal injection of a 1: 1 mixture of chloropromazine 5% and ketamine 2.5% was administered to each rabbit, at a dose of 1 mL/kg animal weight for the 1: 1 mixture, to induce anesthesia with spontaneous respiration. The implantation occurred when the rabbits were asleep, and completely relaxed, without eye movement or response. In the event of any periprocedural reactions, a small amount of the narcotic mixture was re-injected.

The ocular region was cleaned and rinsed with norfloxacin eye drops. The implantation procedure was described by K.E. Wilhelm, previously [11]. We partially modified the procedure as follows: first, a guidewire was introduced into the lacrimal duct slightly from the lacrimal punctum. The hollow stent was positioned using the guidewire. After the stent reached the lacrimal duct, the guidewire was retracted while firmly holding the stent in place to avoid stent dislocation. Finally, the stent was fixed by a stitch in the conjunctiva close to the lacrimal punctum (Fig 2B). After the operation, norfloxacin antibiotic eye drops were applied three times daily for three days.

### Fluorescein excretion and altered stent properties in vivo

At the four time points: 1 week, 4 weeks, 10 weeks and 16 weeks after the implantation, fluorescein excretion test was conducted on all the extant rabbits in Groups A and B. First, three drops of 1% fluorescein solution were applied to the left eye of the rabbit. Dyeing of the mucosa in the nostril of the rabbit by fluorescein 5 min later indicated clear lacrimal duct, and considered positive. In turn, the lacrimal duct was blocked and the experiment was negative in the absence of dying [12].

After the fluorescein excretion experiment, the rabbits in the corresponding groups (A_1_ and B_1_ at 1 week, A_2_, and B_2_ at 4 weeks, A_3_ and B_3_ at 10 weeks, A_4_ and B_4_ at 16 weeks) were sacrificed. The rabbits were anesthetized as described above. The stents were retracted from the lacrimal ducts for further study.

To explore the biodegradability, the stents removed at different time points from the lacrimal ducts were rinsed carefully using double-distilled water three times to remove the secretions and then dried in a vacuum drying box at 35°C for 24 h. Finally, the WLR of the stents was measured and compared with individual pre-implantation data.

### Lacrimal duct endoscopy

As mentioned above, the rabbits in different groups at four time points were anesthetized, and the stents were removed. Subsequently, the rabbits were laid on the operating table and the mucosa was photographed using a lacrimal duct endoscope (Endognost LS200, Polydiagnost GmbH) through the lacrimal punctum anterogradly.

### Histopathological examination of the lacrimal duct epithelium

After the endoscopy, the animals were euthanized by air embolism at each time point in order to collect the lacrimal ducts bilaterally, using the procedure mentioned by Ye et al [13]. First, fluorescein was injected from the lacrimal punctum. The skin from the palpebral fissure to the oral fissure was removed deep into the subcutaneous layer in order to expose the maxilla, premaxilla and the cheek bone. The bone was nibbled open from the port side of the maxilla along with the fluorescein stain used to trace the lacrimal duct. When the entire duct was exposed, it was removed from the bony groove.

The lacrimal duct tissue was vertically split to expose the lacrimal duct epithelium. Histopathological examination was carried out using 10% paraformaldehyde solution, and the tissue was embedded in paraffin for 24 h. The wax blocks were sliced and examined under an optical microscope (Olympus Co., Japan) after hematoxylin and eosin (HE) staining.

### Statistical analysis

The fluorescein excretion results were compared using Fisher probabilities in 2×2 table format. The other data were expressed as mean ± standard deviation (SD). The differences between sample means were analyzed by ANOVA and two-tailed Student-Newman-Keuls method using SPSS version 13.0 (SPSS Inc., Chicago, IL). An alpha value of P < 0.05 was considered statistically significant.

## Results

### Mechanical properties and biodegradability of materials in vitro

**Fig 3:**
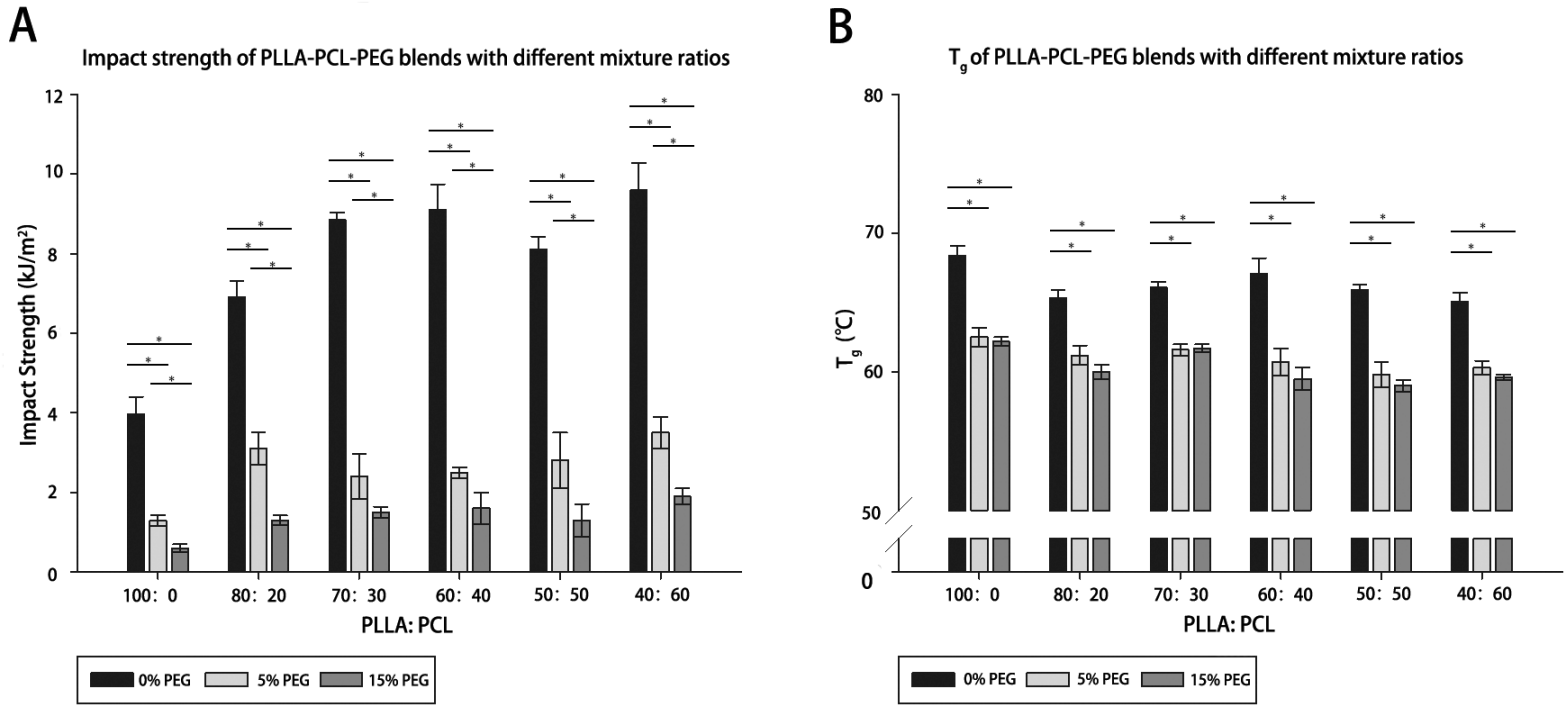
**Diagram A shows the impact strength of PLLA- PCL- PEG blends at different ratios while Diagram B shows T**_**g**_ **of the blends.** Twenty samples of each blend at different ratios were selected randomly for the test. In Diagram A, PCL increased the impact strength of the blends while PEG neutralized the effect. In Diagram B, T_g_ of the blends was decrease by PEG though having nothing to do with the proportion of PEG. The standard deviation is represented by the error bars (P < 0.05). * indicates a statistically significant difference.

PLLA- PCL- PEG complexes showed different mechanical properties corresponding to their ratios. Impact strength was proportional to the toughness of materials, while T_g_ was inversely proportional to the plasticity of materials. PCL increased the toughness of PLLA significantly excluding the effect of PEG (Fig 3A). However, it did not imply that the impact strength was positively correlated with the proportion of PCL. PEG abrogated the impact strength of PLLA- PCL- PEG complexes and neutralized the effect of PCL. The impact strength of the blends decreased further when the proportion of PEG increased from 5% to 15%.

Similarly, PEG showed outstanding plasticity as illustrated in Fig 3B. When mixed with PEG, the T_g_ of the PLLA- PCL complexes decreased significantly, suggesting an increase in plasticity. Nevertheless, the T_g_ of the complex containing 5% and 15% PEG showed no statistically significant difference from each other. Similarly, complexes with different PLLA- PCL ratios did not show significant difference in plasticity under similar PEG levels.

All the complexes degraded and the WLR increased with time (Table 1). However, the degradation rate was a critical challenge. While pure PLLA and PCL degraded slowly, PEG rapidly increased WLR when blended with PLLA and PCL. The proportion of PEG positively correlated with the corresponding WLR. In all the blends, the WLR of PLLA: PCL6: 4+ 15% PEG at Week 16 point was the maximum, suggesting complete degradation. In terms of mechanical properties, the plasticity of PLLA: PCL6: 4+ 15% PEG increased with pure PLLA, without any limitation compared with other blends. Therefore, we concluded that PLLA: PCL6: 4+ 15% PEG stents were the most promising biodegradable stents and selected them for the following study *in vivo*.

**Table 1:** WLR of PLLA- PCL- PEG blends during degradation in vitro.

Stents	Week 4 point	Week 10 point	Week 16 point
	WLR (%, $\bar{x}$±s)		
**PLLA**	0.2 ± 0.1	0.5 ± 0.2	2.2 ± 0.4
**PLLA: PCL = 80:20**	0.0 ± 0.2	-0.2 ± 0.4	1.4 ± 0.4
**PLLA: PCL = 70:30**	-0.2 ± 0.3	-0.4 ± 0.7	1.0 ± 0.3
**PLLA: PCL = 60:40**	0.2 ± 0.4	0.4 ± 1.0	0.7 ± 0.3
**PLLA 5%PEG**	4.6 ± 0.7	8.0 ± 1.2	11.4 ± 1.2
**PLLA: PCL = 80:20 5%PEG**	4.2 ± 1.0	4.5 ± 1.3	6.5 ± 1.1
**PLLA: PCL = 70:30 5%PEG**	4.3 ±1.1	5.4 ± 1.7	8.9 ± 0.9
**PLLA:PCL = 60:40 5%PEG**	7.8 ± 1.1	9.9 ± 2.0	12.0 ± 1.2
**PLLA 15%PEG**	6.6 ± 0.5	8.5 ± 0.4	12.8 ± 0.7
**PLLA:PCL = 80:20 15%PEG**	9.3 ± 1.4	9.9 ± 1.0	13.1 ± 0.5
**PLLA:PCL = 70:30 15%PEG**	8.2 ± 0.7	10.8 ± 1.3	14.6 ± 0.9
**PLLA:PCL = 60:40 15%PEG**	8.6 ± 0.9	10.1 ± 1.1	18.8 ± 0.6

$\bar{x}$, mean. s, standard deviation.

### Altered stent configuration and WLR in vivo

Thirty-two stents (16 PLLA: PCL6: 4+ 15% PEG stent samples and 16 silica gel stents as the control) were successfully implanted in and removed from the lacrimal ducts of the 32 rabbits. The animals tolerated the procedures of implantation and anesthesia. All the animals awoke from narcosis within 2 h and remained alive for the duration of the experiment. During the experimental duration, three rabbits showed superficial purulency at the conjunctiva around the stent implantation site (one in Group B_3_ and two in B_4_), four rabbits showed corneal ulcer (two in Group A_4_ and two in B_4_) and one had ectropion (Group A_4_). The possible reason of superficial purulency was the block of the stents and secondary infection. Corneal ulcer and ectropion probably resulted from the chronic irritation of the stent ends.

No disruption or deformation was found in PLLA: PCL6: 4+ 15% PEG and silica gel stents removed at Week 1. At Week 4 postoperatively, disruption occurred at the ends of the PLLA: PCL6: 4+ 15% PEG stents. However, there was no change in the silica gel stent. At Week 10 postoperatively, the PLLA: PCL6: 4+ 15% PEG stents (Group A_3_) degraded not only at the ends but also in the translucent body, and two of the four samples split into two portions. However, the silica gel stent showed no change. A greater deformation in PLLA: PCL6: 4+ 15% PEG stents occurred at Week 16, including thinning and cracking of tube walls. The configuration disappeared partly and deformed into a thick paste. By contrast, silica gel stents (Group B_4_) showed no disruption or deformation.

The mean values and SD of WLR for A and B groups listed in Table 2 suggest that PLLA: PCL6: 4+ 15% PEG stents cracked and disintegrated *in vivo*. The degradation process accelerated *in vivo* compared with the process *in vitro* and their respective WLRs showed statistically significant difference at Week 10 and 16 points in time after implantation (P < 0.05).

**Table 2:** WLR of the three nasolacrimal stents at the four points in time.

Stent	Week 1 point	Week 4 point	Week 10 point	Week 16 point
	WLR (%, $\bar{x}$±s)			
**PLLA:PCL6:4+15%PEG**	5.44±1.59	6.67±2.53	27.96±4.33	55.08±2.55
**Silica gel**	0.69±1.67	0.30±4.46	-0.53±2.69	2.10±3.53

$\bar{x}$, mean. s, standard deviation.

### Fluorescein excretion

The fluorescein excretion experiment was conducted at the four time points, and the results are shown in Table 3. Using Fisher probabilities in 2×2 table format, we found no statistical difference between the PLLA: PCL6: 4+ 15% PEG group and the silica gel group: the two-tailed P values at Weeks 1, 4, and 10 were 0.71, 0.21 and 0.13, respectively. However, the P at Week 16 was 0.029, which indicated that the PLLA: PCL6: 4+ 15% PEG stents did not obstruct lacrimal ducts as easily as silica gel stents 16 weeks after implantation. The phenomenon was attributed to mucosal secretion at the silica gel stents obstructing the lacrimal ducts. By contrast, the biodegradable stents broke down and cleared the obstructive secretion away from the lacrimal ducts.

**Table 3:** Fluorescein excretion experiment of the three nasolacrimal stents at the four points in time.

Stents	Week 1 point	Week 4 point	Week 10 point	Week 16 point
	Positive/Sample size			
**PLLA:PCL6:4+15%PEG**	14/16	9/12	6/8	4/4
**Silica gel**	15/16	5/12	2/8	0/4

### Lacrimal duct endoscopy

**Fig 4:**
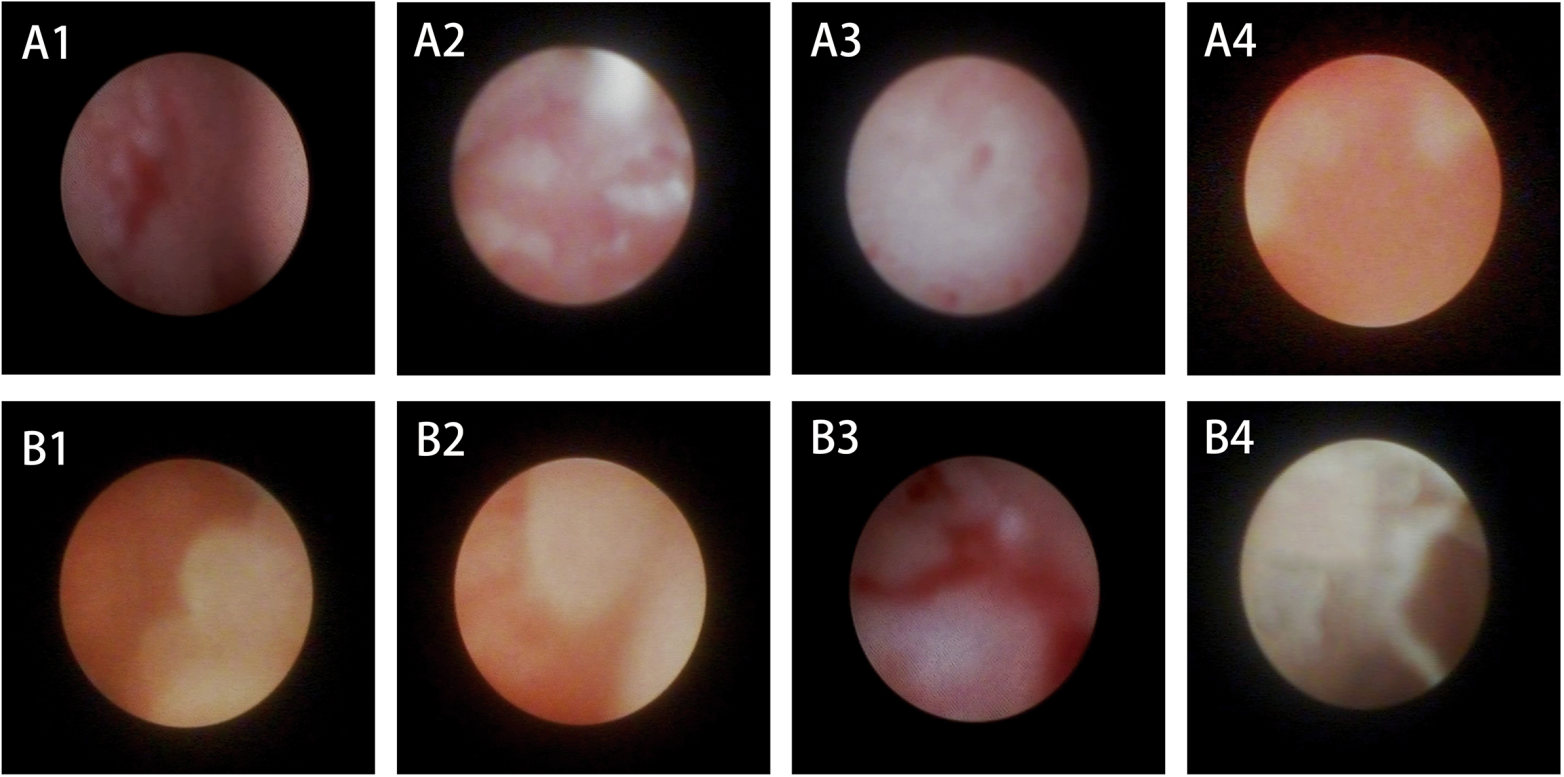
Snapshots of the lacrimal duct endoscopy at four different time points. A1-4 and B1-4 denote the typical endoscopy condition of Group A_1-4_ and B_1-4_. Normal mucosa of the lacrimal duct is rosy and smooth. Hemorrhage (A1) and edema (B1) were common at Week 1 because of acute stimulation by the stents. With time, the mucosa turned red and white, suggesting fibrosis due to inflammation. Papillary epithelial hyperplasia was observed in B3. At Week 16, the mucosa of Group A turned red (A4) while the fibrosis was worse in Group B and the mucosa became pale and rough (B4).

As shown in Fig 4, the lacrimal duct epithelium at Weeks 1 and 4 in Groups A and B showed similar pathological changes: coarse and congestive mucosa with a membrane-like layer covering the epithelium. At Week 10, a white cicatricial tissue appeared in the mucosa of Group A. It was worse in Group B: the cicatricial tissue almost covered the mucosa resulting in polypoid lesions. At Week 16, cicatricial tissue decreased and parts of the mucosa turned smooth due to degradation and breakdown of PLLA: PCL6: 4+ 15% PEG stents. However, the silica gel stents were durable, leading to increased scar formation in the lacrimal epithelium of Group B. The mucosa became rough and nodular.

### Histopathological analysis

**Fig 5:**
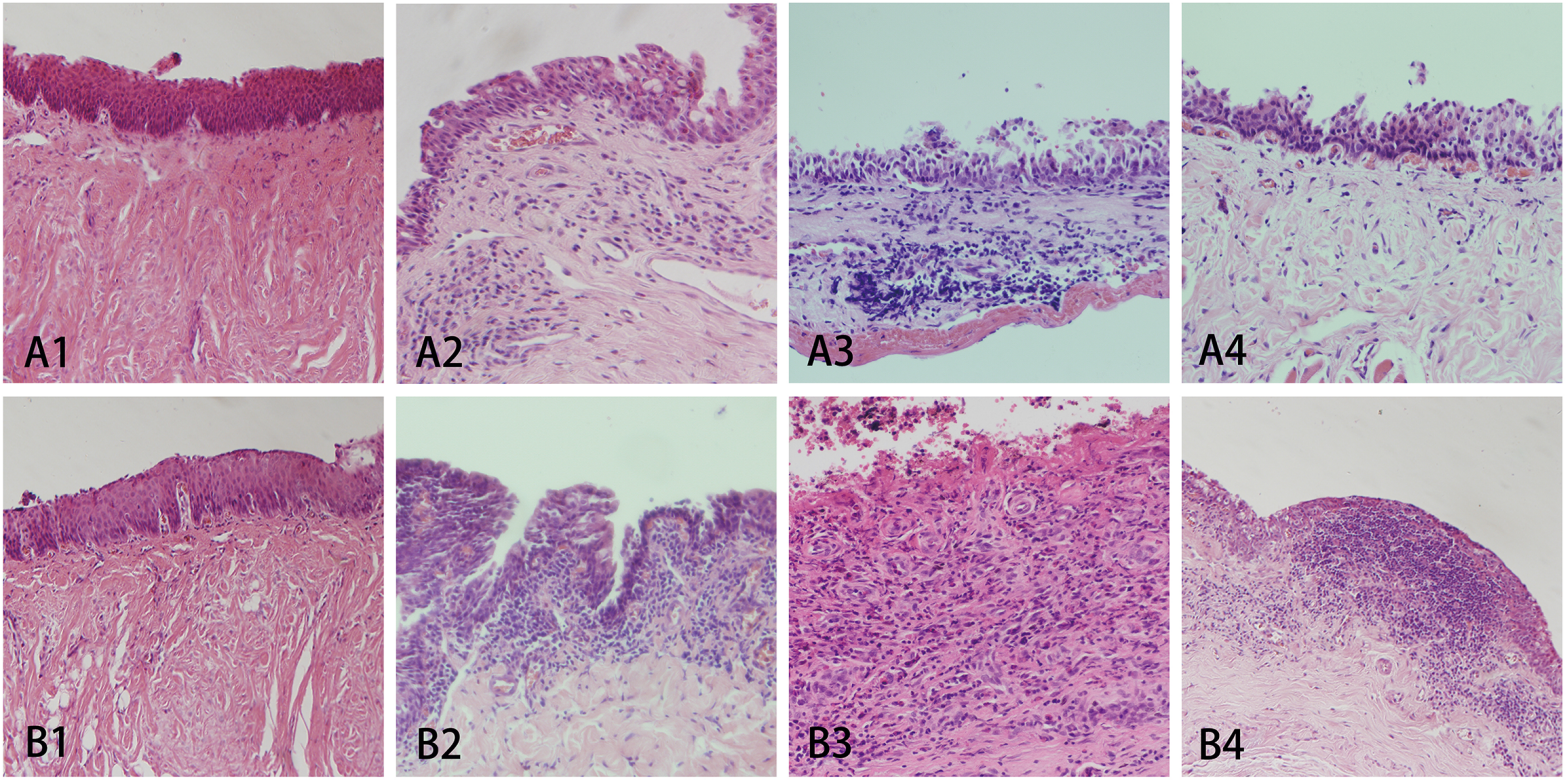
**HE staining of Groups A and B at four different time points.** Similarly, A1-4 and B1-4 represent the typical histopathology of Groups A_1-4_ and B_1-4_. Lumen was located on the upper side of the images. The epithelium, sub-epithelial tissue and muscular layer were located downwards (magnification of A8 was 100× while the other groups were 200×).

HE staining of the lacrimal duct epithelial tissue further confirmed the findings obtained using lacrimal duct endoscopy (Fig 5). At Week 1, no apparent inflammation in the epithelium or sub-epithelium was found, and the stratified epithelium remained intact in Groups A and B. At Week 4, the two groups manifested a more or less severe inflammatory reaction with infiltration of cells into the subepithelial tissue. The epithelial cells showed damage and degeneration with vacuolation. Overall, implantation of the stents led to epithelial damage and inflammation in both Groups A and B, although it was not significantly different between the two groups.

Starting from Week 10, silica gel stents triggered substantial inflammatory reaction due to tissue granulation and incipient subepithelial fibrosis (Fig 5 B3). The epithelial cells were covered by fibrinous exudation. Inflammatory cells extended into the muscular layer and diffused through the lacrimal duct into the adjacent tissue. In case of Group A_3_, the reaction was milder: the basement membrane was intact although the epithelial cells were defective and showed loose epithelial and subepithelial infiltration without apparent subepithelial fibrosis. At Week 16, lymphocytes covered the epithelium completely, and entered the lymphoid nodules, which is an important sign of chronic inflammation in Group B_4_. The inflammatory reaction was ameliorated in Group A_4_, which was characterized by a decrease in inflammatory cell infiltration and restoration of the epithelial cells.

## Discussion

LDOD is a common eye disease. Stent implantation into lacrimal ducts is one of the most effective therapies to re-open the obstruction[14–16]. The stents widely used clinically, such as silica gel stents, show satisfactory early efficacy but lacrimal re-obstruction due to durable irrigation of the stent and consequent subepithelial fibrosis is not rare[17]. Conlon et al reported that chronic tissue damage is a significant feature leading to fibrosis and cicatrization of lacrimal ducts and surgical failure [18]. Surgical removal of stents damages lacrimal epithelium. Therefore, a biodegradable stent, which clears the lacrimal duct and breaks down at an appropriate time, prevents the need for secondary operation.

PLLA was the first material of interest because of biodegradability as well as satisfactory biocompatibility. However, design of hollow stents tailored to the size of lacrimal duct is difficult due to poor mechanical properties of pure PLLA, especially weak plasticity. Furthermore, the biodegradation of pure PLLA was delayed in LDOD suggesting superior degradation, without the risk of re-surgery post-implantation. Subsequently, we introduced two polymers into PLLA, PCL and PEG, to improve mechanical properties of PLLA and accelerate its degradation [19, 20].

Mechanical testing of the blends with different PLLA, PCL and PEG ratios indicated that the addition of PCL and PEG to PLLA directly correlated with toughness and plasticity respectively, while PEG neutralized toughness-increasing effect of PCL. However, it did not imply that the blend with the most satisfactory mechanical properties was the material needed for LDOD treatment since a few blends with high PCL and PEG ratios were not appropriate for the hollow configuration of the lacrimal duct. The degradation range was another important parameter of stent biodegradability. Based on all the parameters, especially, degradation range and WLR, we found that PLLA: PCL6: 4+ 15% PEG, which showed the most satisfactory degradation range *in vitro* was compatible with the classical stent removal time (1 to 3 months) post-implantation. The mucosa was restored and the lacrimal duct regained its function. Meanwhile, the hollow stent shape was optimal in mechanical properties. Therefore, the PLLA: PCL6: 4+ 15% PEG stent was selected for evaluation *in vivo*.

In the *in vivo* study, we administered pure PLLA: PCL6: 4+ 15%PEG stents into the lacrimal ducts of rabbits, and used silica gel stents as the control. As expected, PLLA: PCL6: 4+ 15%PEG stents showed biodegradability *in vivo* and the degradation rate of PLLA: PCL6: 4+ 15%PEG corresponded to the treatment procedure of LDOD. Notably, the PLLA: PCL6: 4+ 15%PEG stents broke down more rapidly *in vivo* than *in vitro*, probably due to the effect of enzymes secreted by the lacrimal duct mucosa, which need further study.

Hollow stent obstruction is a complication associated with stent implantation traditionally because of secretion adhesions, which lead to recurrent epiphora and subsequent removal of the implant. In case of PLLA: PCL6: 4+ 15% PEG stent, sticky lacrimal secretions adhered to the stent, although the lacrimal duct was clear, since the PLLA: PCL6: 4+ 15% PEG stent level diminished continuously. Fluorescein excretion results showed that the fragments of the ends, which attracted the most mucus were most likely disrupted from the main body and left the remaining stent clear.

The lacrimal stent induced epithelial damage in the duct, leading to chronic inflammation and fibrosis of the epithelium and sub-epithelium, and even the muscular layer. The cicatricial tissue narrowed and even clogged the lacrimal duct, suggesting that LDOD recurrence after surgery was not rare[17]. The PLLA: PCL6: 4+ 15% PEG stent fragmented continuously and restored the epithelial cells. The WLR was not similar all along the degradable stent. As the two ends diminished more easily, the WLR of the ends was higher than the calculated value. In terms of the cell size, the degradation rate was high enough to facilitate restoration of the epithelial cells without triggering fibrosis. In turn, the epithelium near the ends of the stents was most susceptible to damage. Therefore, continuous degradation minimized the damage to the epithelium and accelerated cell restoration compared with silica gel stents. The sustained stimulation by silica stents not only enhanced epithelial cell degeneration but also sub-epithelial fibrosis until the stents were removed.

Notwithstanding, the PLLA: PCL6: 4+ 15% PEG stent showed some limits during the study. Firstly, the cracks disintegrated from the stent during the degradation in the lacrimal duct was an underlying hazard for the downstream. White cracks were found flow out with snot from the nostrils of the rabbits after the degradable stent implantation. It was possible that big crack would stuck at stenosis and cause secondary lacrimal duct obstruction. Though downstream lacrimal duct obstruction had not been examined in our study, we were unsure about the absence in studies with more samples or other animals. Inflammation was another problem. The PLLA: PCL6: 4+ 15% PEG stent gave rise to less lacrimal duct inflammation compared with the silica gel stent, but it was not enough for a desirable lacrimal duct stent, because chronic inflammation caused by the stents was one of the most significant factors leading to recurrence of LDOD after the therapy of stent intubation[4].

While PCL and PEG increased the degradation rate of pure PLLA stent and blends at the various ratios tested, we still could not conclude that PLLA: PCL6: 4+ 15% PEG was the optimal composition for lacrimal stent. Inferred from Table 1, complexes with higher proportions of PEG, in theory, show shorter degradation range which meant less inflammation reactions and complications, such as superficial purulency at the conjunctiva around the stent implantation site and corneal ulcer. But single screw extrusion failed to generate complexes containing PEG higher than 15% as well as complexes of PLLA- PCL 50:50 and 40:60 with PEG 5% and 15%. We could just test samples on hand and concluded limited result. Meanwhile, we tested the degradation of PLLA: PCL6: 4+ 15% PEG stent alone *in vivo* instead of all the complexes tested *in vitro* due to funding constraints and manpower shortage. Nonetheless, according to our study, PLLA: PCL6: 4+ 15% PEG stent was an acceptable lacrimal biodegradable stent for LDOD, though not necessarily the best.

In summary, the study results suggest that the PLLA: PCL6: 4+ 15%PEG stent is a satisfactory biodegradable stent, which showed tissue compatibility, biodegradation and adequate mechanical intensity to prevent the re-obstruction of lacrimal duct. Therefore, the PLLA: PCL6: 4+ 15%PEG stent is a promising alternative for therapeutic application in LDOD.
